# Psychiatric patients’ attitudes towards being hospitalized: a national multicentre study in Norway

**DOI:** 10.1186/s12888-022-04362-8

**Published:** 2022-11-21

**Authors:** Kjetil Hustoft, Tor Ketil Larsen, Kolbjørn Brønnick, Inge Joa, Jan Olav Johannessen, Torleif Ruud

**Affiliations:** 1 Center of Clinical Research in Psychosis, Department of Adult Psychiatry, Stavanger University Hospital, 8100, Jan Johnsen gate 12, N-4068 Stavanger, Norway; 2 Department of Clinical Medicine, University of Bergen, Bergen, Norway; 3 Department of Public Health, Faculty of Health Sciences, University of Stavanger, Stavanger, Norway; 4 Centre of Age-related Medicine, Stavanger University Hospital, Stavanger, Norway; 5 Network for Medical Sciences, Faculty of Health, University of Stavanger, 4036 Stavanger, Norway; 6 Division of Mental Health Services, Akershus University Hospital, Lørenskog, Norway; 7 Institute of Clinical Medicine, University of Oslo, Oslo, Norway

**Keywords:** Psychiatry, Involuntary hospitalization, Attitudes, Acute psychiatric wards, Mental health legislation, Coercion, Insight, Autonomy, Capacity to consent, Mental capacity

## Abstract

**Background:**

The aim of the study was to explore patients’ attitudes towards voluntary and involuntary hospitalization in Norway, and predictors for involuntary patients who wanted admission.

**Methods:**

A multi-centre study of consecutively admitted patients to emergency psychiatric wards over a 3 months period in 2005–06. Data included demographics, admission status (voluntary / involuntary), symptom levels, and whether the patients expressed a wish to be admitted regardless of judicial status. To analyse predictors of wanting admission (binary variable), a generalized linear mixed modelling was conducted, using random intercepts for the site, and fixed effects for all variables, with logit link-function.

**Results:**

The sample comprised of 3.051 patients of witch 1.232 (40.4%) were being involuntary hospitalised. As expected 96.5% of the voluntary admitted patients wanted admission, while as many as 29.7% of the involuntary patients stated that they wanted the same. The involuntary patients wanting admission were less likely to be transported by police, had less aggression, hallucinations and delusions, more depressed mood, less use of drugs, less suicidality before admission, better social functioning and were less often referred by general practitioners compared with involuntary patients who did not want admission. In a multivariate analysis, predictors for involuntary hospitalization and wanting admission were, not being transported by police, less aggression and less use of drugs.

**Conclusions:**

Almost a third of the involuntary admitted patients stated that they actually wanted to be hospitalized. It thus seems to be important to thoroughly address patients’ preferences, both before and after admission, regarding whether they wish to be hospitalized or not.

## Background

Patients’ lack of insight in their mental illness is a challenge and may interfere with patients’ willingness for admission [1]. Patients often deny being ill despite obvious symptoms such as psychosis, mania or severe depression [2–4]. This stands in sharp contrast to somatic medicine where patients with severe symptoms usually want admission and demand treatment.

The Norwegian Mental Health Care Act gives physicians the right to admit a patient for involuntary hospitalization (IH) when a major psychiatric illness is present, represents a danger to self or others, and the patient denies the need for treatment. Voluntary mental health care has to have been attempted, or have to be deemed futile, for instance if the patient lacks the capacity to give informed consent.

Voluntary mental health care has been tried, to no avail, or it is obviously pointless to try this [5]. From 2002 to 2006, the use of IH in Norway ranged from 36 to 44%, including admissions in geronto- and forensic psychiatry [6–8]. There is an extremely wide range of reported levels of IH. In other Scandinavian countries, rates of IH have been reported to vary from 4.6% in Denmark, to 30% in Sweden, and in Europe (1990–2000) with a range from 3.2% in Portugal to 44.8% in Germany [9, 10]. Many studies are on selected samples and methodology is often unclear.

Hospitalization is influenced by several stakeholders such as the patients themselves, the caregivers, GPs and health personnel, physicians at municipal emergency clinics, other people in the social network in which the patient is embedded, socio-political context, the media, or the general public’s attitude towards psychiatry. Factors such as access to health care, availability of treatment, diagnostic evaluation, use of psychiatric medication, economic costs, and the quality of the psychiatric facilities also influence the use of IH [11–16]. To be IH may increase stigma of having a mental disorder for example by prejudice that patients are dangerous and less competent, and patients may feel discriminated as a group [17].

In the last decade, there has been increased focus on the use of IH. The United Nations Convention on the Rights of Persons with Disabilities (CRPD) is an international treaty that identifies the rights of persons with disabilities as well as the obligations for States parties to promote, protect and ensure those rights [18]. The main purpose of the CRPD is to ensure that disabled people have equal opportunity to realize their human rights and to reduce obstacles that make this difficult. It has been argued that it is the interest of psychiatry to reduce its reliance on coercion and implement alternative ways of support for the psychiatric patient [19]. In Norway politicians have decided that use of IH should be reduced despite lack of research on what is a reasonable level of IH [20]. In a study from Norway, 2001 people were interviewed by telephone by an independent polling company about IH on their views on use of coercion in psychiatry. Between 87 and 97% strongly or partial agreed with the use of IH when they were presented specific case-examples [21].

In the present study, we had the opportunity to ask at intake a large sample of 3.051 consecutively admitted psychiatric patients whether they actually wanted to be admitted or not. The aim of the study was to explore patients’ attitudes towards voluntary and involuntary hospitalisation in Norway, and predictors for involuntary patients who wanted admission.

Based upon a review of the literature our hypothesis was that the majority of voluntary hospitalized patients (VH) would state that they wanted to be admitted [22]. As we see it, the question of what IH patients would experience, is more open.

## Methods

### Design

This is a cross-sectional multi-centre study of a large cohort of patients consecutively admitted to psychiatric emergency wards in Norway during the fall 2005 and spring 2006.

### Sample

Admission data were collected from all hospitalizations during 3 months at 20 psychiatric emergency units [23]. The health trusts included all geographical regions and 75% of all psychiatric emergency wards in Norway. We identified 3.338 cases. Due to missing data regarding whether they wanted admission or not, 3.051 cases were included in the study. The involuntary hospitalized (IH) group included patients admitted for compulsory observation up to 10 days (section 3–2 in the Mental Health Care Law), or compulsory mental health care (section 3–3 in the Mental Health Care Law), and a small number of patients under other law paragraphs (chapter 5 in the Mental Health Care Law - court order for transfer to compulsory mental health care, and Law of Child Protection and Law of Social Services) [5].

### Measures

We collected the following sociodemographic data: age, gender, ethnicity, having children under the age of 18, childcare status, housing status, source of income, educational level and services received prior to admission. We recorded admission time and date, whether this admission was acute or elective, referral agency, legal status - voluntary or involuntary, whether transported to the hospital by police and previous contact with mental health agencies [23]. All patients were asked whether they wanted to be hospitalized or not.

Functioning was measured by the Global Assessment of Functioning (GAF) split version scale of axis IV in DSM-IV, with symptoms (GAF-S) and functional level (GAF-F) scored separately on a scale from one to 100. Higher scores indicated less symptoms and better functioning [24–27].

Psychiatric problems were measured by the 12-item Health of the Nation Outcome Scales (HoNOS) for behaviour, cognitive impairment, symptoms and social functioning. The scale used the following scores; zero (no problem), one (minor problem which do not need action), two (mild problem but definitely present), three (moderately severe problem) and four (severe to very severe problem) [28, 29].

Drug and alcohol abuse for the 6 month prior to admission was assessed by the Alcohol and Drug Use Scale being; zero (abstinent), one (use without impairment), two (abuse), three (dependency), and four (dependency requiring institutionalization) [25, 30, 31].

### Data collection and procedure

Psychiatric nurses, nurses, nurse assistants, resident physicians, psychiatrists and clinical psychologists carried out the data collection. Health personnel participated in local training sessions regarding use of the Admission Registration Form, developed for this study, through discussions and scoring vignettes [32]. The Admission Registration Form was completed by the clinician treating the patient or other health professionals participating in the patient’s admission to the ward. The admission form did not record the date of assessment or the name of the assessor. Data were deidentified, and transferred to a central database.

### Statistics

For descriptive statistics, frequencies, means and standard deviations (SD) were calculated. A binary variable representing wanting admission [1] or not (0) was the outcome variable in the analyses using generalized linear mixed modelling, using random intercepts for the site to correct for different base-rates at the different sites, and fixed effects for all variables, with logit link-function. All effects were presented as odds-ratios (OR) with corresponding 95% confidence intervals. Individual analyses were performed for each variable in order to estimate the unadjusted effects. All variables showing unadjusted significant effects on wanting admission were entered simultaneously in the GLIMMIX procedure to estimate adjusted multivariate effects. Analyses were carried out with the use of SPSS 22.0 [33] and the GLIMMIX module of SAS Academic version 3.3 was used for generalized linear mixed modelling [34].

The study was approved by the Regional Ethical Committee in Eastern Norway (reg. no. 04049) and the Norwegian Social Science Data Service and The Norwegian Data Inspectorate under the Norwegian Ministry of Labour and Government Administration, NSD (reg. no. 11074).

## Results

Altogether 3.051 patients were included, 40.4% of them were involuntary hospitalized (IH). Of all patients, 69.5% stated they wanted to be admitted. The majority of voluntary hospitalized (VH) wanted admission (96.5%). In the IH group, we found that almost one-third (29.7%) stated the same (Table 1).

**Table 1:** Proportion of voluntarily and involuntary hospitalized patients who stated they wanted or did not want admission

	Voluntary hospitalized	Involuntary hospitalized	Total sample
	n (%)	n (%)	N (%)
**Wanted admission**	1755 (96.5)	366 (29.7)	2121 (69.5)
**Did not want admission**	64 (3.5)	866 (70.3)	930 (30.5)
**Sum**	1819 (100.0)	1232 (100.0)	3051 (100.0)

The IH patients wanting admission were less likely to be transported by police, had less aggression, hallucinations and delusions, more depressed mood, less use of drugs, less suicidality before admission, better social functioning and less referred by general practitioners compared with involuntary patients who did not want admission (Table 2).

**Table 2:** Socio-demographic and clinical characteristics of involuntary hospitalized patients who stated that they wanted or did not want admission

	Involuntary hospitalized patients							
		Wanted admission			Did not want admission			
	N	Mean	n (%)	S.D.	Mean	n (%)	S.D.	sig*
**Demographics**								
Age	1230	39.3	365 (29.7)	14.6	40.7	865 (70.3)	17.0	0.172
Gender; male	1230		211 (57.5)			461 (53.4)		0.169
Country of origin -Norwegians	1216		321 (88.7)			755 (88.4)		0.992
Marital status	1202							
Unmarried			218 (60.4)			496 (59.0)		0.654
Married/divorced/ separated/widowed			143 (39.6)			345 (41.0)		
College or university	1133		56 (16.5)			115 (14.5)		0.530
Living situation, living alone	1126		203 (60.1)			439 (55.7)		0.189
**Admission process**								
Referring agent	1062							
GP			61 (19.1)			178 (24.0)		0.047
Emergency primary health care clinic			156 (48.9)			377 (50.7)		
From psychiatric health care			102 (32.0)			188 (25.3)		
Referral source did not know the patient	1225		226 (62.4)			547 (63.4)		0.795
Transported by police	1185		109 (30.8)			511 (61.5)		< 0.001
No previous contact with psychiatric services	1181		91 (25.9			218 (26.3)		0.942
Admission, evening and night versus daytime	1195		272 76.2)			600 (71.6)		0.125
**Symptoms**								
GAF at intake								
Symptoms	1203	34.1	359	11.5	31.0	844	12.4	< 0.001
Function	1203	36.6	359	12.4	34.2	844	11.9	< 0.001
HoNOS								
Overactive, aggressive or agitated behaviour	1172	1.16	350	1.25	1.56	822	1.38	< 0.001
Non-accidental self-injury	1166	0.99	355	1.40	0.83	816	1.36	0.056
Hallucinations and delusions	1162	1.65	348	1.46	1.92	814	1.49	0.004
Depressed mood	1159	1.50	349	1.28	1.20	810	1.25	< 0.001
Appeared intoxicated at admission	1218		39 (3.2)			103 (8.5)		0.319
Use of drugs (score 3–5; misuse, dependency, need for institutionalization)	1211		116 (9.6)			207 (17.1)		0.015
Suicidal danger before admission	1228		159 (43.6)			438 (50.7)		< 0.001
Suicidal danger in psychiatric ward (moderate or high)	1132		56 (15.7)			103 (13.3)		0.146
Patient fulfilled a suicide attempt during hospitalization	1217		6 (1.7)			17 (2.0)		0.820
Patient did self-harm during hospitalization	1216		15 (4.2			51 (6.3)		0.267
Patient did physical attack on others during hospitalization	1222		28 (7.7)			98 (11.4)		0.149
Patient was physical attacked by others during hospitalization	1219		4 (1.1)			11 (1.3)		0.869

*S.D * Standard Deviation

*p - value significant < 0.05

In a multivariate analysis, we found that being IH and wanting admission was predicted by being less often transported by police, having less aggressive and agitated behaviour and less use of drugs (Table 3).

**Table 3:** Predictors for patients involuntary hospitalized who stated that they did want admission^a^

IH and wanted admission	*P* - value	Odds ratio	95% confidence interval (C.I.)
**Referring agent**			
Local out-of-office-hours casualty clinic	0.193	0.984	0.664–1.457
General practitioner (GP)	ref	1.330	0.866–2.045
From psychiatric health care	0.215	1.353	0.839–2.181
Transported by police	0.000	0.272	0.194–0.381
**Symptoms ratings at admission**			
GAF-S symptoms at intake	0.332	1.008	0.992–1.025
GAF-F functioning at intake	0.566	1.005	0.988–1.022
HoNOS aggression	0.050	0.880	0.763–1.000
HoNOS hallucinations and delusions	0.469	0.953	0.837–1.086
HoNOS reduced mood level	0.066	1.149	0.991–1.332
drugs	0.000	1.263	1.117–1.429
suicidal danger	0.880	0.990	0.870–1.127

*IH* Involuntary Hospitalized, *GAF* Global Assessment of Functioning (Function and Symptoms), *HoNOS* Health of the Nation Outcome Scale

^a^ = GLIMMIX module of SAS Academic version 3.3 was used for generalized linear mixed modelling

## Discussion

We found that nearly one third of IH patients and 96.5% of VH patients stated that they wanted to be hospitalized when asked after they were admitted to a hospital.

Two studies and a review of outcome studies have reported similar results. In a study from the USA of 260 consecutively admitted patients they found that 52.6% IH group stated that they needed hospitalization, and 85.9% of the VH group stated the same [22]. An English mixed method follow up study of 778 IH patients from 22 rural and urban hospitals reported patients’ attitudes to IH within the first week of hospitalization [35]. One year after discharge, 96 patients were re-interviewed. Patients with higher level of functioning at baseline were less likely to consider their IH as justified compared to patients with lower level of functioning. Patients who were less satisfied with treatment the first week of IH reported the index IH admission as less justified. The rate of IH patients who wanted hospitalization was not described at intake. However, 40% of IH patients interviewed after 1 year felt their admission was justified. A qualitative study with a subsample of 59 of these patients found that on admission, 25.4% of IH patients felt that the hospitalization was necessary [36].

Based on a review article of 18 outcome studies of IH, three of the studies interviewed IH patients within the first 25 days after admission [37]. Between 39 and 58% of the IH patients stated that hospitalization was needed. However, these studies were rated as to a low to median level of quality, and they focused on changes of attitudes at follow up rather than what characterizes patients at admission.

How can we understand this seemingly counter intuitive finding? There are many dilemmas related to this kind of research. It would be expected that voluntary hospitalized (VH) patients would state that they wanted admission, and that IH did not want admission. However, studies have shown that patients are not always aware of whether they are voluntary or involuntary hospitalized. In a Norwegian study they found that 41% of IH patients believed they were on voluntary status, while 32% of VH patients thought they were on involuntary status [38].

In primary health care, there might be a tradition for using IH when the physician is unsure of whether the patient is psychotic or suicidal. In specialised care, there might be lack of beds leading to an increased threshold for acute admissions. Lack of less restrictive alternative forms of care has been shown to be associated with more use of IH [15].

In a Norwegian study about attitudes towards IH of different stakeholder (former patients, relatives of patients, member of supervisory committees, psychiatrists, other physicians and lawyers), psychiatrist and physicians were in more favour of using IH for patients who were unable to care of themselves, harm themselves or others, compared to the other groups [39].

The reasons why physicians outside the psychiatric hospital level want to admit patients involuntary could be many, including that the physician may have been uncertain about whether the patient would stay voluntarily in the hospital, discharge himself / herself and then harm self or others due to an unstable mental health status. The physician may be afraid to make a serious mistake. The use of IH could be a final safeguard for the physician. Physicians at a municipal emergency primary health care clinic have limited time to evaluate symptoms and put up a list of pros and cons for an IH, and perhaps might not have explored and listened carefully to the patient’s opinions regarding wanting hospitalization or not. Often the physicians do not know the patients well [40, 41]. Physicians may feel concerned about being criticized by health authorities for evaluating the patients wrongly and therefore select IH to be on the legally safe side [41]. Cultural or traditional aspects may interfere as well. A study of informal coercion in 10 countries indicate that mental health care professionals work with ambivalence and contradictory expectations [42].

In Norway, the Mental Health Care Act has a section 3–4 that prevents the transmission from voluntary to involuntary admission once the patient is admitted [43]. A voluntary hospitalized patient has the right to discharge himself / herself anytime if not in danger for self or others The VH patient may not be converted to compulsory observation or compulsory mental health care. However, the prohibition in the first paragraph does not apply in cases where discharge means that the patient constitutes an obvious and serious risk to his or her own life and health and those of others. Very few cases in Norway are converted from VH to IH (201 in 2018) [44]. In some countries, they do not have such prohibition of conversion from VH to IH. In Denmark (2001) the proportion of IH adult persons in relation to the total number of psychiatric inpatients admitted that year were 7.1% [45]. However, in Denmark, the same year, the proportion of forcibly detained patients within the hospital (converted from VH to IH after maximum 7 days of admission in hospital) was 8.1%. This shows that Danish Mental Health Care Law has a more open possibility to take care of the uncertainty GPs may have, without discharging the patient and then readmit the patient on an IH status.

For the patient, there might be changes in attitudes towards being hospitalized during the admission phase. Some studies have focused on the IH admission process from the patient point of view. The IH patients felt frightened, overwhelmed, confused and experienced a loss of control in the admission process. There were also concerns of disrupted family relationships [46, 47]. IH patients wished health personnel had more focus on contact with patients, closeness, and understanding. They wanted personnel to wait instead of acting. Physicians highlighted the importance of human contact and mutual relationship in the hospital setting to prevent coercion [47]. For family caregivers, the most common response to admission was relief, worry and guilt, and frustration over delays of getting help in acute settings [48].

In our study, IH patients who said that they wanted admission had a better mental health state with better global functioning, fewer used drugs and evaluated with less suicidal danger before admission. However, they had a higher score on depression. In the multivariate analysis the factor regarding depression did not receive significance as a predictor (Table 3). These results are all descriptions of IH patients with less severe psychiatric symptoms, and - we could presume - with a better insight. However, this findings are in contrast to results were IH patients in retrospect who justified their admission had a lower level of global functioning at admission [35].

The police are the only agency with the right to use force against individuals outside the psychiatric hospital [49]. The police are only needed when patients are aggressive and have to be secured and prevented from harming self or others. This corresponds with our results that predictors of IH patients who wanted admission were; less transported by police, less aggressive and agitated behaviour and less likely to use drugs. Overall, IH patients who wanted admission may not have been in need for police assistance due to their better behaviour and not affected by use of illegal drugs.

As expected, in our study almost all VH patients wanted admission. The results seems to confirm that VH patients agreed it was a correct decision by the GP to admit them. However, 3.5% of VH said they did not want admission. We did not have a follow up question that could explain this finding. However, some VH patients have reported in several studies that being admitted to a psychiatric emergency unit in itself feels like a coercion [50–54]. Our findings might also mean that IH are too often used in Norway since almost one third stated that they wanted to be hospitalized. Maybe GPs ought to use more time and investigated more profoundly the patient’s opinion of admission in a dialogue during the consultation and a tighter discussion with the hospital if IH is the best solution for the patient.

In Norway, there has been a legal adjustment with the introduction of consent competence in the new Mental Health Care Act from the autumn of 2017. Nevertheless, admission to compulsory mental health care (IH) is not reduced during the last years. At the Norwegian national level, the number of referrals for IH seems very similar in 2016 (11.939) to 2018 (11.783). There are no calculations for the last 4 years [55].

### Strengths and limitations

The major strength of this study was a large and representative sample of consecutively admitted patients. The inclusion of patients did not depend on letter of consent from the patients. Thus, all cases were included in the study. In Norway, we have a national psychiatric health care system free of charge, and no acute private health care system. The inclusion of cases did not depend on consent from the patients.

The group of missing patients (287 cases) who did not answer if they wanted or did not want admission did not differ from the rest on major characteristics such as gender, age, use of drugs and general symptom levels (HoNOS).

Limitations were that we had multiple raters and locations with no possibility to carry out a reliability test between all raters. There could also be a delay until when the raters asked the question of wanting admission or not during the admission process, since we did not have registration of when the question was asked, and what kind of health professionals who asked the question.

## Conclusions

Almost a third of involuntary admitted patients stated that they wanted admission. This raises serious questions about the practice around admission of involuntary referred patients, representing a possible threat to the patients’ autonomy. A basis for a future dialogue about alternative ways of dealing with the patient’s serious mental condition could be by using more time, more in-depth ask what options the patient could imagine for developing a positive admission by preserving the patient’s autonomy and co-determination. As a result, there could be a reduction in unnecessary involuntary hospitalization and reduced burden on the health service in processing such admissions.

## Data Availability

The dataset is stored in a de-identified format at Department of adult psychiatry, Stavanger University Hospital, Stavanger Norway, and is available from the corresponding author on reasonable request.

## Abbreviations

VH: Voluntary hospitalization;
IH: Involuntary Hospitalized
GAF: Global Assessment of Functioning (Function and Symptoms)
HoNOS: Health of the Nation Outcome Scale
S.D.: Standard Deviation
NSD: Norwegian Social Science Data Service
